# Microbial Consortium Application Under Temperature Stress: Effects on the Rhizosphere Microbiome and Plant Growth

**DOI:** 10.3390/ijms262411814

**Published:** 2025-12-07

**Authors:** Ekaterina Alexeevna Sokolova, Natalya Valentinovna Smirnova, Valeria Aleksandrovna Fedorets, Inna Viktorovna Khlistun, Olga Viktorovna Mishukova, Irina Nikolaevna Tromenschleger, Oleg Aleksandrovich Savenkov, Oleg Igorevich Saprikin, Evgeny Ivanovich Rogaev, Maria Dmitrievna Buyanova, Irina Mikhailovna Filippova, Taisiya Maksimovna Mayorova, Marina Andreevna Glukhova, Mitina Maria Ivanovna, Andrey Dmitrievich Manakhov, Elena Nikolaevna Voronina

**Affiliations:** 1 Institute of Chemical Biology and Fundamental Medicine, Siberian Branch of the Russian Academy of Sciences, 630090 Novosibirsk, Russia; 2 Department of Natural Sciences, Novosibirsk State University, 630090 Novosibirsk, Russia; 3 Institute of Soil Science and Agrochemistry, Siberian Branch of Russian Academy of Sciences, 630090 Novosibirsk, Russia; 4 Department of Genetics, Centre for Genetics and Life Science, Sirius University of Science and Technology, 354340 Sirius, Russia

**Keywords:** synthetic microbial consortium, PGPR, rhizosphere microbiome, metagenomic analysis, agricultural crops, chernozem, gray forest soil, abiotic stress

## Abstract

The aim of the present study was to investigate the effect of a synthetic microbial consortium (SMC) containing five functionally different bacterial strains (*Rahnella aquatilis*, *Rothia endophytica*, *Stenotrophomonas indicatrix*, *Burkholderia contaminans*, *Lelliotia amnigena*) on the growth and development of three agricultural crops (wheat, buckwheat, and rapeseed) on two soil types (chernozem and gray forest soil) under field conditions. The experiment was conducted from June to September 2024 under extreme field conditions, with temperatures reaching 43.8 °C. This study evaluates SMC efficacy under severe abiotic stress, reflecting increasingly common climate extremes. Metagenomic data analysis showed that the introduced strains did not establish stable populations in the soil, possibly due to heat-induced bacterial mortality, though other factors including competition with indigenous microflora and lack of protective formulations may have also contributed. No statistically significant effects on plant morphometric parameters were observed. The extreme temperature and water stress conditions appear to have been the dominant limiting factors, overriding any potential benefits from microbial inoculation, as evidenced by the lack of response to mineral fertilizer application as well. Crop-specific effects were revealed: when cultivating rapeseed on chernozem, a significant increase in available phosphorus content was noted (from 278 ± 45 to 638 ± 92 mg/kg with SMC application, *p* < 0.001).

## 1. Introduction

The use of growth-promoting rhizospheric bacteria, also known as plant growth-promoting rhizosphere bacteria (PGPR), is a promising approach for sustainable agriculture [1,2]. This approach reduces the need for chemical fertilizers and pesticides, but the transition from laboratory studies to field applications can be challenging due to the difficulty in maintaining the viability of introduced strains in competitive soil environments [3,4,5].

The creation of synthetic microbial consortium (SMCs), which combine strains with complementary functional characteristics, is considered a way to enhance the effectiveness of microbial preparations [6,7]. The diverse functional abilities of the consortium are expected to provide more stable benefits for plants through various mechanisms, such as nitrogen fixation, the solubilization of poorly accessible phosphorus, the production of plant hormones and siderophores, and biocontrol of pathogenic microorganisms [6,8]. However, the success of the SMC application depends not only on the functional characteristics of the strains included in it, but also on the ability of the microorganisms in the consortium to establish stable populations in the rhizosphere and interact effectively with the native soil microbiome [9,10]. Additionally, PGPR efficacy depends strongly on edaphic and climatic conditions, which can vary significantly in the field [11].

Climate change is increasing the frequency of extreme weather events, including heat waves and droughts, which pose challenges for both crop production and PGPR implementation [12,13,14]. High temperatures (>40 °C) can negatively affect bacterial survival through membrane damage, protein denaturation, and metabolic disruption [15], while simultaneously imposing severe physiological stress on plants [16,17]. Most PGPR field studies have been conducted under moderate conditions or with supplementary irrigation [18,19], but there is growing recognition that PGPR performance under naturally occurring extreme stress needs better characterization to develop realistic expectations for field applications under climate change conditions.

Western Siberia has a sharply continental climate with cold winters and warm summers, where temperature fluctuations and periodic summer heat waves create challenging conditions for agriculture. The region contains diverse soil types including fertile chernozems and gray forest soils with varying fertility levels. The short growing season (approximately 100–120 days) and variable precipitation patterns require crops to develop rapidly and cope with fluctuating environmental conditions. Indigenous soil microbial communities in these soils have adapted to extreme seasonal temperature variations and may present strong competition for introduced bacterial strains.

The present study was conducted in Western Siberia to evaluate the effect of a synthetic microbial consortium (SMC) containing five functionally complementary bacterial strains (*Rahnella aquatilis*, *Rothia endophytica*, *Stenotrophomonas indicatrix*, *Burkholderia contaminans*, *Lelliotia amnigena*) on three agricultural crops (wheat, buckwheat, and rapeseed) grown on two soil types (chernozem and gray forest soil) under field conditions. During the 2024 growing season, the experimental site experienced unusually high temperatures (up to 43.8 °C) and limited precipitation, which provided an unplanned opportunity to assess SMC performance under extreme abiotic stress. The objectives were to evaluate: (1) the establishment and persistence of introduced strains in the rhizosphere; (2) SMC effects on plant growth parameters; (3) changes in the indigenous rhizosphere microbiome structure using metagenomic analysis; and (4) the interaction between SMC, mineral fertilizers, soil type, and crop species under these field conditions.

## 2. Results

### 2.1. The Physico-Chemical Properties of the Soils

Two types of soils most commonly found in agriculture in Western Siberia were selected for this study: chernozem and gray forest soil. These soils differ significantly in their physical and chemical properties (Table 1). Gray forest soils have a neutral pH level (7.1 ± 0.2) compared to chernozems, which have a higher pH (7.8 ± 0.1). They also have lower levels of soluble salts (specific electrical conductivity (SEC): 83.1 ± 0.8 μs/cm vs. 206.1 ± 0.9 μs/cm) and lower concentrations of organic carbon and total nitrogen content (C_org: 7.2 ± 0.2% vs. 10.3 ± 0.4%, N Total: 0.5 ± 0.1% vs. 1.5 ± 0.01%). Available mineral nitrogen, phosphorus, and potassium are also lower in gray forest soil compared to chernozems (N-NO_3_: 3.5 ± 0.3 mg/kg vs. 4.5 ± 0.7 mg/kg, P_2_O_5_: 1.2 ± 0.9 mg/kg vs. 11.3 ± 1.2 mg/kg, K_2_O: 98 ± 0 mg/kg vs. 139.5 ± 2.1 mg/kg).

### 2.2. Introduction of Microbial Consortium Strains in Soil

Metagenomic data analysis showed that introduced microbial consortium strains were not detected in the soil in significant quantities either in July or September. The relative abundance of corresponding bacterial genera/species in treated variants did not differ from control samples without microbial consortium application (*p* > 0.05 for all comparisons). This indicates that a single application of liquid consortium suspension at the time of sowing proved insufficient to establish a stable bacterial population in the rhizosphere. This result is critical for interpreting all subsequent observations, as it demonstrates that the observed effects are related not to the direct action of living cells of introduced strains, but rather to changes in the indigenous microbiome and/or to the influence of metabolites from dead microbial consortium cells.

**Figure 1:**
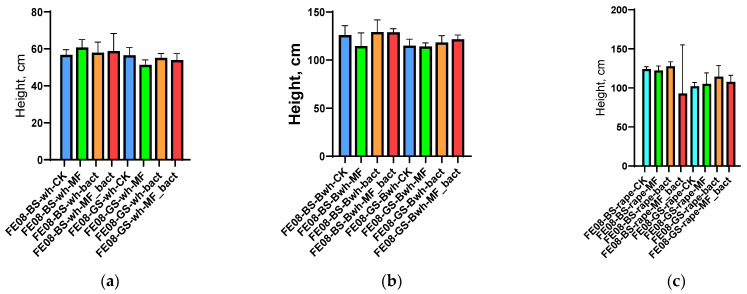
Plant morphometric indicators: (**a**) wheat height; (**b**) buckwheat height; (**c**) rapeseed height. BS—chernozem, GS—grey soil, CK—without treatment, MF—mineral fertilizers, bact—microbial consortium, MF_bact—microbial consortium combined with mineral fertilizers, wh—wheat, Bwh—buckwheat, rape—rapeseed.

Application of microbial consortium, mineral fertilizers, and their combination did not lead to statistically significant changes in morphometric parameters for any of the studied crops (*p* > 0.05 for all comparisons). Average plant height was 35.2 ± 4.1 cm (control) vs. 36.8 ± 3.9 cm (microbial consortium) for wheat, 28.5 ± 3.2 cm vs. 29.1 ± 3.7 cm for buckwheat, and 42.1 ± 5.3 cm vs. 43.2 ± 4.8 cm for rapeseed (Figure 1). NPK content in plant tissues also did not differ between different treatment variants (Supplementary Figures S1−S6).

A similar pattern was observed for plant biomass parameters. Aboveground dry mass did not differ between treatment variants for all crops. Interestingly, in the case of buckwheat on chernozem, a slight, albeit statistically insignificant, decrease in growth parameters was observed with mineral fertilizer application compared to the control, but this effect did not manifest in treatments with bacterial inoculation or bacteria combined with fertilizers.

The content of major nutrient elements (N, P, K) in plant tissues also did not differ between treatment variants (*p* > 0.05), indicating no improvement in mineral nutrition of plants in the current growing season.

### 2.3. Agrochemical Soil Parameters at the End of the Growing Season

By the end of the growing season, no significant differences in the content of major nutrient elements were found between variants with and without microbial consortium application for the same soil and crop (*p* > 0.05). However, interesting differences were observed between different plants.

**Figure 2:**
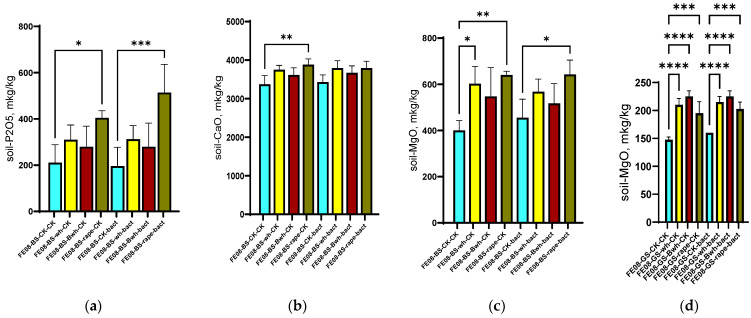
Agrochemical soil parameters at the end of the growing season: (**a**) Available P in chernozem; (**b**) Ca content in chernozem; (**c**) Mg content in chernozem and (**d**) Mg content in dark forest soil, columns 1–4 without treatment, columns 5–8 with microbial consortium treatment. BS—chernozem, GS—grey soil, CK—without treatment, bact—microbial consortium, wh—wheat, Bwh—buckwheat, rape—rapeseed. Significance level: *—*p* < 0.05, **—*p* < 0.01, ***—*p* < 0.001, ****—*p* < 0.0001.

On chernozem, a significant increase in available phosphorus content was revealed when cultivating rapeseed (from 278 ± 45 mg/kg in the control to 512 ± 83 mg/kg under rapeseed cultivation, *p* < 0.001). Furthermore, this difference was enhanced with microbial consortium application (638 ± 92 mg/kg, *p* < 0.001 compared to unplanted control) (Figure 2a). Rapeseed cultivation resulted in an increase in soil calcium (from 2850 ± 320 to 3640 ± 280 mg/kg, *p* < 0.01) (Figure 2b) and magnesium (from 425 ± 65 to 580 ± 75 mg/kg, *p* < 0.05) (Figure 2c). Interestingly, for gray forest soil, an increase in magnesium content was observed for all plant species (from 95 ± 12 to 155–185 mg/kg, *p* < 0.001), which most likely corresponds to magnesium accumulation in the upper layer from decomposed organic matter (plant “litter”). On gray forest soil, this difference was detectable for all plants due to its initially low soil content. The increase in available phosphorus occurs on phosphorus-rich chernozem and is significant only for rapeseed, which possibly indicates either the secretion of certain root exudates promoting phosphate solubilization or specific interactions with soil bacteria, which in turn actively promote phosphate solubilization.

### 2.4. Structure of Soil Microbial Communities

#### 2.4.1. Alpha-Diversity

**Figure 3:**
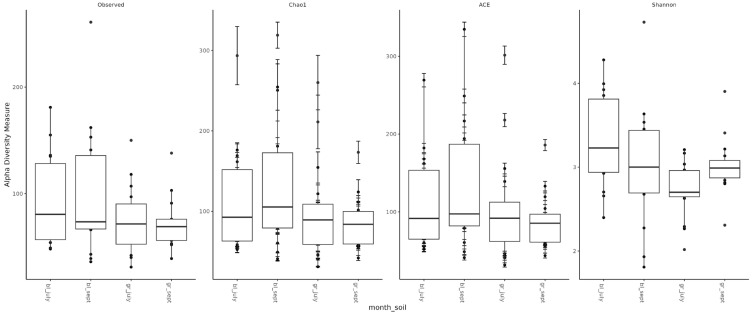
Alpha-diversity of soil microbial communities depending on soil type and study month. bl—chernozem, gr—gray forest soil, sept—September, july—July.

**Figure 4:**
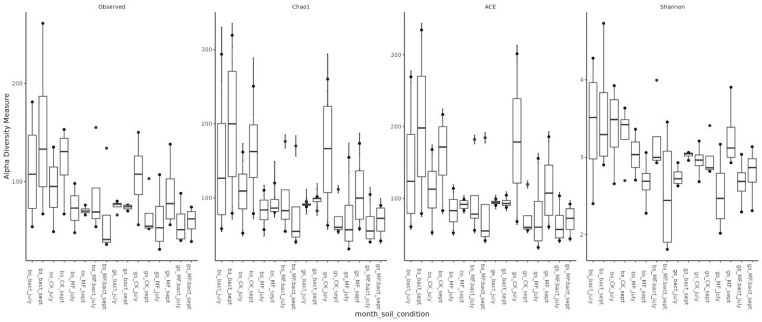
Alpha-diversity of soil microbial communities depending on soil type, study month, and treatment method. Bs—chernozem, gs—grey soil, sept—September, CK—without treatment, MF—mineral fertilizers, bact—microbial consortium, MF_bact—microbial consortium combined with mineral fertilizers.

Assessment of microbial diversity showed that, as expected, diversity in chernozem soil is higher than in gray forest soil (Figure 3). When analyzing the effect of treatment on microbiome diversity, the following observations can be made: (1) In chernozem, microbial consortium application slightly increased microbial diversity, while mineral fertilizer application led to a small but statistically significant decrease. (2) For gray forest soil, a similar trend can be traced, but it is less pronounced. Interestingly, with microbial consortium treatment in gray forest soil, stabilization of the alpha-diversity indicator is observed (reduced variability between replicates), while for chernozems, conversely, the spread of values between different plants increased (Figure 4).

#### 2.4.2. Beta-Diversity

**Figure 5:**
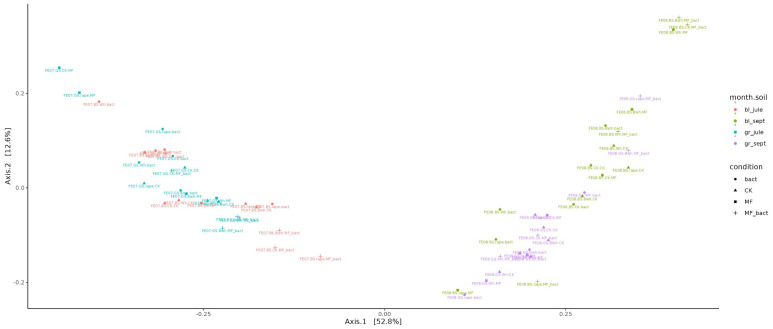
Visualization of beta-diversity data using Bray–Curtis distance metric.

Beta-diversity assessment showed that the factor of temporal succession of the microbial community had the greatest influence on microbiome composition (Figure 5). The ordination diagram shows a clear separation into two groups: July and September samples (the first principal component PC1 explains 52.8% of variability, *p* < 0.001). At the same time, no significant difference is observed between gray forest soil and chernozem (PC2 explains 12.3% of variability). It can only be noted that in September, the microbiomes of gray forest soil and chernozems cluster better than in July. Also, no clear patterns are observed in changes of overall microbial diversity between different treatments and different plants.

#### 2.4.3. Taxonomic Composition of Microbial Communities

**Figure 6:**
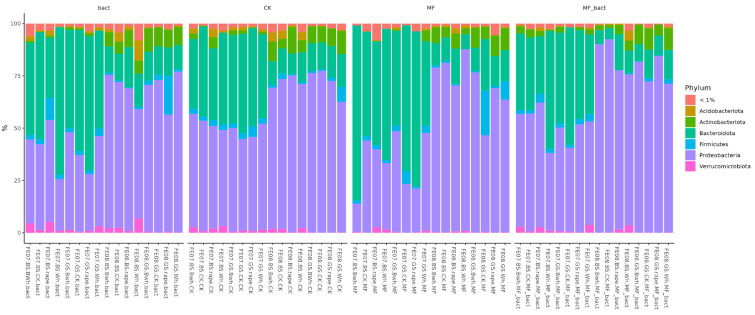

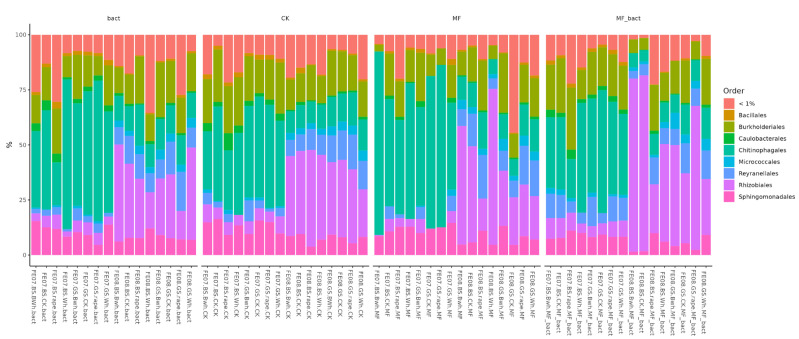
Abundance bar plot by phylum and order levels. BS—chernozem, GS—grey soil, CK—without treatment, MF—mineral fertilizers, bact—microbial consortium, MF_bact—microbial consortium combined with mineral fertilizers, wh—wheat, Bwh—buckwheat, rape—rapeseed, FE07-metagenome analysis in July, FE08-metagenome analysis in September.

Analysis of the microbiome at the phylum level shows two main trends (Figure 6). The first is a decrease in the representation of *Proteobacteria* with soil treatment. The second is an increase in the representation of *Proteobacteria* by the end of the growing season. These changes were observed independently of treatment type and crop, indicating their connection to general seasonal factors.

If we pay attention to the order level, it is striking that the main trend in seasonal succession is an increase in the abundance of the order *Rhizobiales*. Moreover, in control plantings this occurs consistently and uniformly across all soil types and plants, while with fertilizer application it begins to vary greatly. Subsequently, we conducted a more detailed analysis of the influence of soil/plant/fertilizer interactions on the microbiome specifically at the order level.

**Figure 7:**
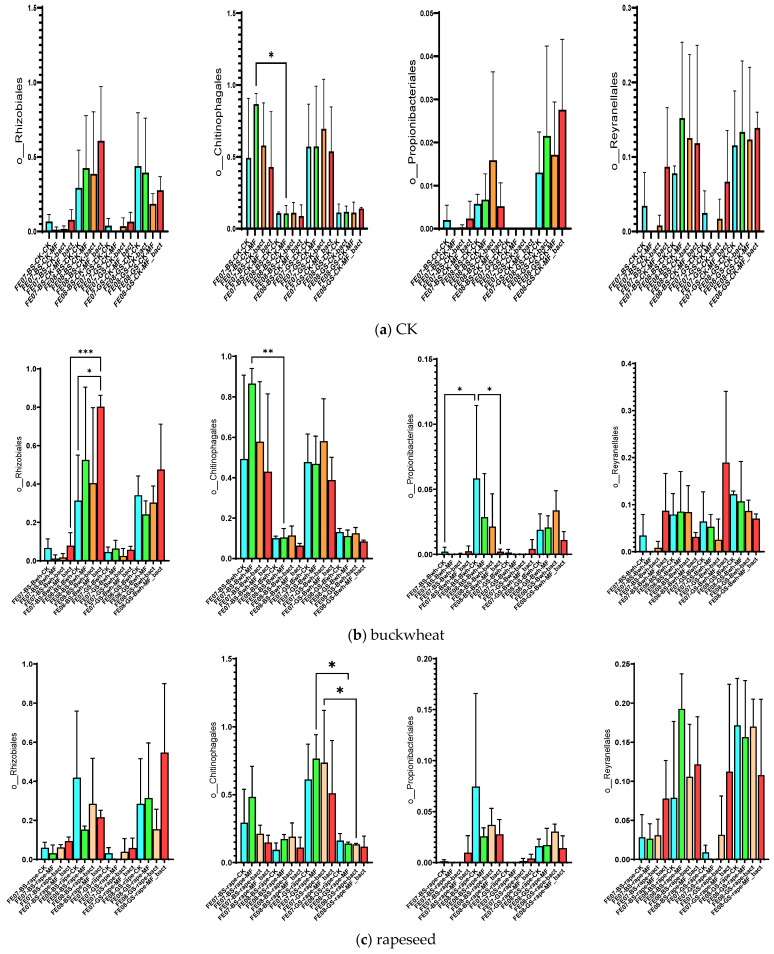

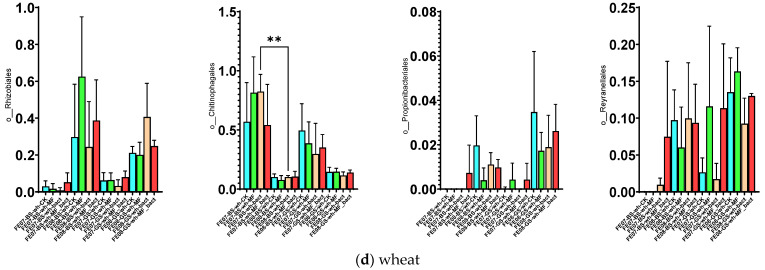
Proportion of representatives of a particular order among all representatives of the metagenome. FE07-metagenome analysis in July, FE08-metagenome analysis in September, BS—chernozem, GS—gray forest soil, CK—without treatment, MF—mineral fertilizers, bact—microbial consortium, MF_bact—microbial consortium combined with mineral fertilizers, (**a**) without plants, (**b**) buckwheat, (**c**) rapeseed, (**d**) wheat. Significance level: *—*p* < 0.05, **—*p* < 0.01, ***—*p* < 0.001.

By the end of the growing season (September), an increase in the relative abundance of bacteria of the orders *Rhizobiales*, *Propionibacteriales*, and *Reyranellales* was observed in all plants and fertilizer treatments, as well as a decrease in the representation of *Chitinophagales* (Figure 7). These changes reflect the microbial community’s adaptation to changing conditions: accumulation of plant residues, changes in soil moisture and temperature, and depletion of readily available substrates.

*Rhizobiales* represent an ecologically diverse group, including not only symbiotic nitrogen-fixing bacteria of legumes, but also numerous free-living soil bacteria capable of degrading complex organic compounds [20,21]. Their increase toward the end of the growing season may be associated with the accumulation of root exudates and plant litter. *Propionibacteriales*, belonging to *Actinobacteria*, are often associated with the decomposition of recalcitrant organic compounds and may play a role in late stages of plant residue degradation [22]. The decrease in *Chitinophagales*, which specialize in the degradation of chitin and other polysaccharides, may reflect the depletion of these substrates by the end of the season [23].

It is important to note that these temporal trends manifested independently of soil type and crop, indicating their universal nature and connection to general seasonal factors.

#### 2.4.4. Specific Effects of SMC Application at the Level of Bacterial Orders

**Figure 8:**
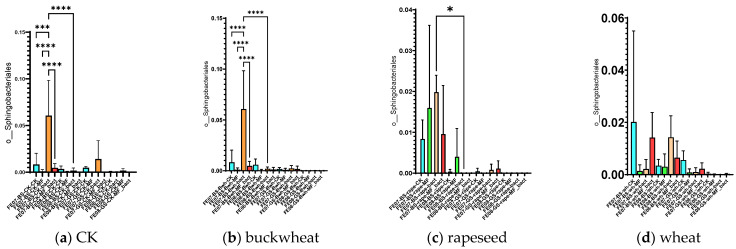
The proportion of representatives of the order *Sphingobacteriales* relative to total metagenome. FE07-metagenome analysis in July, FE08-metagenome analysis in September, BS—chernozem, GS—gray forest soil, CK—untreated, MF—mineral fertilizers, bact—SMC, MF_bact—SMC together with mineral fertilizers, (**a**) without plants, (**b**) buckwheat, (**c**) rapeseed, (**d**) wheat. Significance level: *—*p* < 0.05, ***—*p* < 0.001, ****—*p* < 0.0001.

The introduction of SMC led to some temporary changes in the composition of the native microbial community. The most notable effect was a significant increase in the proportion of *Sphingobacteria* (mainly represented by the genus *Pedobacter*) on chernozem in July with SMC application (an increase from 0.015 ± 0.005 to 0.065 ± 0.012, *p* < 0.001). However, this effect did not persist by September (Figure 8).

*Pedobacter* are Gram-negative, aerobic bacteria that are widely distributed in soils and can degrade a variety of organic substances, including complex polysaccharides and aromatic compounds [24]. Their temporary increase may be attributed to utilization of cellular components from lysed SMC cells or metabolites produced prior to cell death in the first few weeks after application. This phenomenon is consistent with the “priming effect”—a mechanism whereby addition of labile organic matter temporarily stimulates specific microbial groups [18]. In this case, the introduced bacterial cells likely died rapidly under extreme temperature conditions, and their biomass and metabolites served as readily available substrates that stimulated indigenous bacteria capable of rapid substrate utilization, particularly *Sphingobacteriales* such as *Pedobacter* [17]. This interpretation is supported by the absence of detectable introduced strains and the transient nature of these changes.

**Figure 9:**
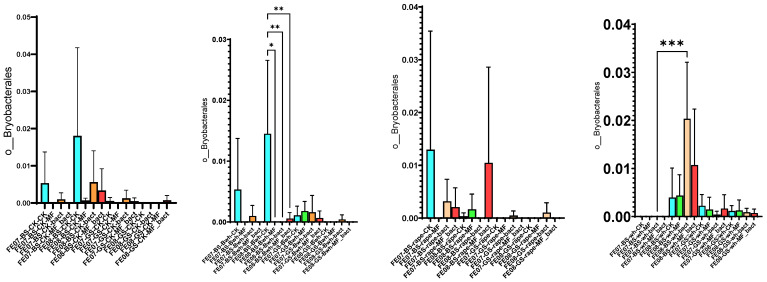

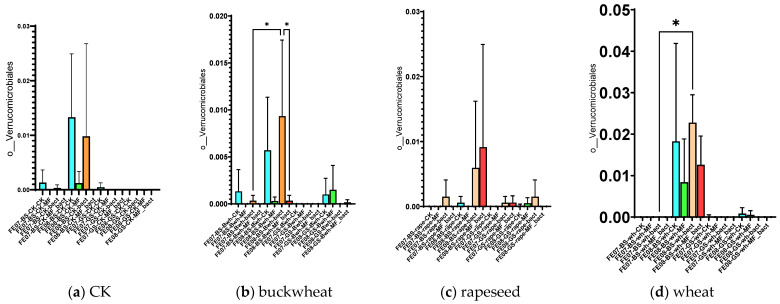
The proportion of representatives of the *Bryobacterales* and *Verrucomicrobiales* orders from all representatives of the metagenome. FE07-metagenome analysis in July, FE08-metagenome analysis in September, BS—chernozem, GS—gray forest soil, CK—untreated, MF—mineral fertilizers, bact—SMC, MF_bact—SMC together with mineral fertilizers, (**a**) without plants, (**b**) buckwheat, (**c**) rapeseed, (**d**) wheat. Significance level: *—*p* < 0.05, **—*p* < 0.01, ***—*p* < 0.001.

By September, *Bryobacterales* and *Verrucomicrobiales* showed increased abundance in untreated chernozem (Figure 9). This increase was partially suppressed when mineral fertilizers were applied. SMC application also had an intermediate effect, but suppressed these taxa less than mineral fertilizers of these taxa compared to mineral fertilizers. *Verrucomicrobiales* and *Bryobacterales* are often found in oligotrophic conditions, where there is a slow growth rate (K-strategy) [25,26]. The increased abundance of these taxa in untreated soil may indicate that the microbial community was transitioning to nutrient-limited conditions.

#### 2.4.5. Crop-Specific Changes

Various agricultural crops create specific conditions in the rhizosphere due to the composition and quantity of root exudates, the architecture of the root system, and other factors. These conditions lead to the formation of specific microbial communities that are unique to each crop.

##### Buckwheat

**Figure 10:**
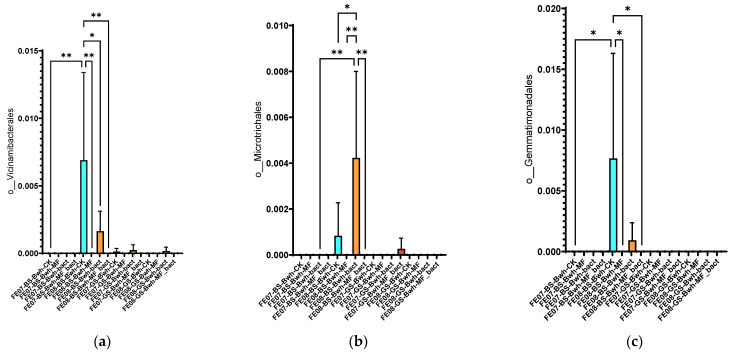
The proportion of representatives of certain orders from all representatives of the metagenome. FE07-metagenome analysis in July, FE08-metagenome analysis in September, BS—chernozem, GS—gray forest soil, CK—untreated, MF—mineral fertilizers, bact—SMC, MF_bact—SMC combined with mineral fertilizers, (**a**) *Vicinamibacterales*, (**b**) *Microtrichales*, (**c**) *Gemmatimonadales*. Significance level: *—*p* < 0.05, **—*p* < 0.01.

Soil under buckwheat showed the most significant changes in the microbial community (Figure 7b). There was an increase in the abundance of *Rhizobiales* and *Propionibacteriales*, which was more pronounced than in the control plots without plants. This increase was especially notable with combined MF+SMC treatment (an increase of *Rhizobiales* from 0.35 ± 0.08 in the control to 0.82 ± 0.12, *p* < 0.001). At the same time, *Reyranellales* did not exhibit an increasing trend, in contrast to the control plots. Changes in the order of *Vicinamibacterales*, *Microtrichales*, and *Gemmatimonadales* were also specific to buckwheat and showed an increase by September (*p* < 0.05) (Figure 10). Interestingly, the introduction of SMC enhanced the effect on *Microtrichales* and *Verrucomicrobiales*, whereas mineral fertilizers seemed to suppress it.

Significant differences in the case of the SMC application were observed in July for *Sphingobacteriales*, mainly due to the genus *Pedobacter*, similarly to control samples without plants (Figure 8).

##### Rapeseed

The microbial community under rapeseed differed from the control and buckwheat communities (Figure 7c). *Rhizobiales* accumulated less than in buckwheat but more than in the control, while *Propionibacteriales* increased and *Reyranellales* increased similarly to the control by the end of the growing season. *Chitinophagales* tended to decrease on gray forest soils only.

**Figure 11:**
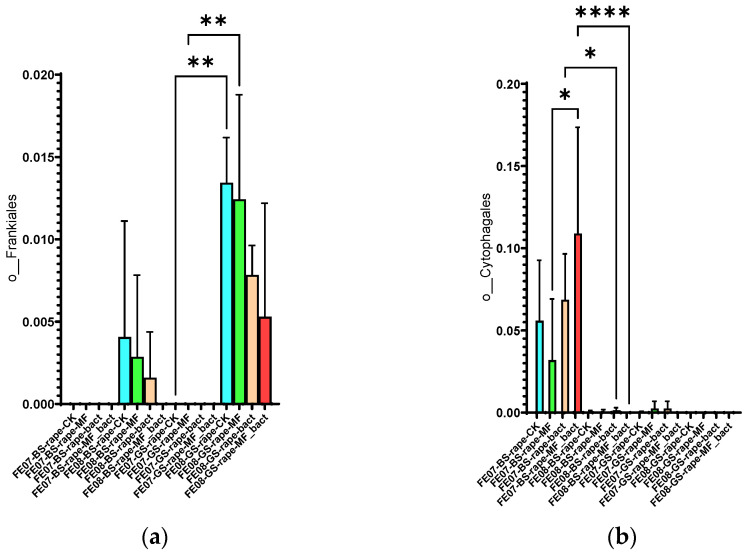
The proportion of representatives of the orders *Frankiales* and *Cytophagales* from all representatives of the metagome. FE07-metagenome analysis in July, FE08-metagenome analysis in September, BS—chernozem, GS—gray forest soil, CK—untreated, MF—mineral fertilizers, bact—SMC, MF_bact—SMC together with mineral fertilizers, (**a**) *Frankiales*, (**b**) *Cytophagales*. Significance level: *—*p* < 0.05, **—*p* < 0.01, ****—*p* < 0.0001.

*Frankiales* significantly increased in rapeseed by September, especially on gray forest soil, from 0.002 ± 0.001 to 0.015 ± 0.003 (*p* < 0.01). *Cytophagales* decreased on chernozem from 0.095 ± 0.018 to 0.045 ± 0.012 (*p* < 0.001) by the same period (Figure 11). The accumulation of *Frankiales* may be related to their ability to degrade organic compounds typical of rapeseed root exudates [27,28]. Rapeseed is known to produce glucosinolates and isothiocyanates that have a selective effect on soil microbiota.

No significant increase was observed in the number of *Sphingobacteria* in July after the introduction of SMC. Additionally, in the case of rapeseed, there was not a significant increase in the numbers of *Bryobacterales*, *Verrucomicrobiales*, *Vicinamibacterales*, *Microtrichales*, and *Gemmatimonadales* by September.

##### Wheat

The microbial community under wheat showed an intermediate position between the control and buckwheat for most indicators. The accumulation of *Rhizobiales* was lower than that of buckwheat, while *Reyranellales* showed a slight increase, intermediate between buckwheat and the control. By the end of the growing season, *Chitinophagales* bacteria tended to decrease only on gray forest soils (*p* < 0.01) (Figure 7d).

No wheat-specific taxonomic shifts were identified. This may be due to the relatively neutral effect of wheat on the soil microbiota, compared with buckwheat and rapeseed. This agrees with previous reports showing that cereal crops typically have a less pronounced effect on the rhizosphere microbial community [29].

## 3. Discussion

### 3.1. The Mechanism of SMC Influence on the Microbial Community

A critical observation of the present study is the absence of significant quantities of introduced strains in the soil both in July and September. This indicates that a single application of liquid bacterial consortium suspension (10^8^ CFU/mL) was insufficient for rhizosphere colonization. Metagenomic analysis revealed no significant enrichment of bacterial genera corresponding to the introduced strains (*Rahnella*, *Rothia*, *Stenotrophomonas*, *Burkholderia*, *Lelliotia*) in SMC-treated plots compared to control plots at either sampling time point. These genera were detected at low, baseline levels across all treatments, consistent with the presence of indigenous members of these taxa in the native soil microbiome. Probable causes include: (1) high competition from indigenous microflora [9]; (2) extreme temperature conditions (up to 43.8 °C) leading to rapid mortality of introduced cells; (3) absence of protective formulations ensuring strain survival (alginate beads, peat-based carriers) [30,31].

The observed changes in the indigenous microbiome are therefore related not to the direct action of viable bacterial consortium cells, but to a short-term “priming effect”: metabolites and components of dead bacterial consortium cells stimulated certain groups of indigenous bacteria [32]. The most pronounced effect was observed on *Sphingobacteriales* (genus *Pedobacter*) in July on chernozem soil, which is consistent with the known ability of these bacteria to rapidly utilize readily available organic substrates [24]. This supports the concept of the “priming effect”, whereby the addition of labile organic matter stimulates the activity of specific soil microbial groups.

The transient nature of these changes (disappearance of the effect by September) confirms the hypothesis that they were driven by short-term utilization of components from the introduced bacteria rather than establishment of stable interactions between introduced strains and plants [33].

### 3.2. No Effects on Plants

The absence of effects on plant morphometric parameters, despite changes in the microbiome and some agrochemical shifts, can be explained by three main factors:

#### 3.2.1. Extreme Climatic Conditions as the Dominant Limiting Factor

Air temperatures reaching 43.8 °C in the absence of supplementary irrigation beyond natural precipitation induced multiple stresses in plants: water deficit, photosynthetic apparatus damage, membrane permeability disruption, and oxidative stress [34]. PGPR can partially mitigate the negative effects of drought and heat stress through various mechanisms: production of osmoprotectants (proline, glycine betaine), induction of plant antioxidant systems, improvement of water status through modulation of root architecture, and production of exopolysaccharides [35]. However, the strains comprising the current bacterial consortium either did not possess sufficient stress-protective activity or, most likely, failed to establish effective interaction with plants before the onset of peak temperature stress due to rapid cell death.

Critical confirmation that climatic conditions, rather than nutrient deficiency, were the limiting factor is the fact that even the application of a half-dose of mineral fertilizers did not lead to improvement in plant morphometric parameters [36]. Moreover, in the case of buckwheat on chernozem soil, a slight decrease in parameters was observed with mineral fertilizer application compared to the control. This observation could theoretically be explained by the fact that under severe water deficit, the addition of mineral fertilizers increases solute concentration in the limited soil solution, lowering its osmotic potential and creating an additional barrier to root water uptake.

Thus, the absence of positive effects from both bacterial consortium and mineral fertilizer applications indicates that water and temperature stress were the overriding limiting factors in this experiment. Under such extreme conditions, nutritional interventions—whether biological or chemical—could not compensate for the severe physiological constraints imposed by drought and heat stress.

#### 3.2.2. Ineffective Rhizosphere Colonization and Inability to Realize PGPR Mechanisms

The absence of introduced strains in the soil indicates that the potential plant growth-promoting mechanisms of the bacterial consortium (nitrogen fixation, phosphate solubilization, phytohormone production) could not be realized in practice. Literature evidence demonstrates that successful rhizosphere colonization often requires repeated applications or the use of formulations that ensure prolonged bacterial release (e.g., alginate beads, peat-based carriers) [31,37]. Moreover, manifestation of stress-protective effects requires establishment of effective plant-bacteria interactions prior to stress onset, which in our experiment may not have occurred due to the rapid onset of peak temperatures and death of the introduced strains.

Short-term changes in the indigenous microbiome (increased *Sphingobacteriales* in July) proved insufficient to significantly affect plants. The transient nature of these changes and their association with the priming effect did not provide long-term improvement in soil microbial community functioning.

### 3.3. Temporal Factors and Potential Delayed Effects

Changes in microbial community structure and agrochemical parameters (particularly the increase in available phosphorus under rapeseed by the end of the growing season) may have occurred too late in the season to affect already established plants. For example, the increase in available phosphorus under rapeseed from 278 ± 45 to 638 ± 92 mg/kg by September may not have had time to influence the current crop but potentially creates more favorable conditions for the subsequent crop in rotation.

Similarly, shifts in the microbial community observed in September may affect organic matter mineralization processes and nutrient release during the autumn-winter and following spring periods [38]. This underscores the importance of multi-year studies: effects of microbiological preparations are often cumulative and manifest not immediately but with repeated applications and under favorable conditions.

### 3.4. Crop-Specific Effects and Their Mechanisms

Different agricultural crops created specific rhizosphere conditions, leading to crop-specific changes in the microbiome and agrochemical parameters.

Buckwheat showed the most pronounced changes in microbial community composition with increases in representatives of various functional groups (*Rhizobiales*, *Vicinamibacterales*, *Microtrichales*, *Gemmatimonadales*). These findings align with Japanese studies by Morigasaki et al. (2024), which identified 870 bacterial taxa in buckwheat-cultivated andosols, with characteristic enrichment of *Burkholderiales* during maturation stages [39,40]. Interestingly, bacterial consortium application enhanced the growth of oligotrophic groups (*Microtrichales*, *Verrucomicrobiales*), while mineral fertilizers suppressed them, maintaining a more copiotrophic state of the microbial community.

The pronounced response of the buckwheat rhizosphere microbiome can be attributed to the distinctive composition of buckwheat root exudates, particularly the secretion of flavonoids and phenolic compounds. Buckwheat (Fagopyrum esculentum) produces and exudes substantial quantities of flavonoids, including rutin, quercetin, and other phenolic compounds [40]. These compounds serve multiple functions: they act as carbon sources for specific bacterial groups, function as signaling molecules that selectively stimulate certain rhizosphere bacteria, and can modulate microbial community assembly [41]. Recent work by Wu et al. (2025) [34] demonstrated that specific buckwheat flavonoids (6,7,4′-trihydroxyisoflavone and 7,3′,4′-trihydroxyflavone) actively shape microbial communities, creating a “microbial legacy” effect not observed in cereal crops.

Rhizobiales, in particular, are known to respond to flavonoid compounds through specific signaling pathways that can trigger various metabolic functions including those involved in nutrient cycling and organic matter decomposition [42]. The ability of flavonoids to chelate metal ions and modulate soil pH may additionally create favorable conditions for certain bacterial groups. The combination of SMC application with buckwheat’s naturally rich and diverse exudate profile likely created conditions favorable for specific bacterial groups, resulting in the most pronounced microbiome shifts observed among the three crops studied.

For rapeseed, the most significant effect was the increase in available phosphorus content on chernozem soil, which was enhanced by bacterial consortium application. This result is consistent with multi-year studies on rapeseed microbiomes showing dominance of *Proteobacteria* (30–56%), *Actinobacteria* (15–33%), and *Bacteroidetes* (5–14%), with phosphate-mobilizing bacteria (*Bacillus megaterium*, *B. subtilis*, *Pseudomonas fluorescens*) demonstrating 21–44% yield increases in field trials [43]. Research on gray forest soils in Russia [44] specifically documented that ACC-deaminase bacterial consortium explained 73% of microbiome variation in rapeseed versus only 24% for mineral fertilizers, increasing *Proteobacteria* by 38% and correlating with seed yield (r = 0.85, *p* < 0.01).

The distinctive microbiome structure observed in the rapeseed rhizosphere is likely shaped primarily by the production of glucosinolates and their hydrolysis products, isothiocyanates (ITCs). Glucosinolates are sulfur-containing secondary metabolites characteristic of Brassicaceae species, which upon tissue damage or through root exudation are hydrolyzed by myrosinase enzymes to release ITCs and other breakdown products [45]. These compounds possess antimicrobial properties and exert strong selective pressure on the soil microbial community, suppressing sensitive taxa while favoring ITC-tolerant microorganisms [46,47].

The observed accumulation of Frankiales and decrease in Cytophagales in the rapeseed rhizosphere reflects this selective pressure. Frankiales, which include many actinobacterial taxa, are often associated with stress tolerance and possess the metabolic capacity to degrade complex aromatic compounds [21,22], potentially conferring resistance to glucosinolate derivatives. Studies have demonstrated that Brassicaceae plants can shape soil microbiota community structure to favor bacteria and fungi adapted to glucosinolates and ITCs [46]. This selective recruitment may represent a co-evolutionary adaptation whereby rapeseed establishes beneficial microbial consortia capable of thriving in the unique chemical environment created by glucosinolate exudation.

Furthermore, the breakdown products of glucosinolates can influence nutrient cycling, particularly phosphorus mobilization, through effects on soil pH and stimulation of specific microbial groups involved in phosphate solubilization [48]. The significant increase in available phosphorus observed in our study by September may result from this complex interaction between glucosinolate-mediated microbiome restructuring and enhanced activity of phosphate-mobilizing bacteria. However, the timing of this phosphorus increase—occurring late in the growing season—suggests that the benefits may be more pronounced for subsequent crops in rotation rather than for the current rapeseed crop.

Wheat occupied an intermediate position for most parameters, which is consistent with literature data indicating that cereal crops typically exert less pronounced selective pressure on the rhizosphere microbial community compared to legumes and *Brassicaceae*. Previous studies have demonstrated that wheat varieties show relatively limited effects on rhizosphere microbiome composition and diversity compared to the substantial influence of soil type and environmental factors [29,49]. Although wheat produces specific root exudates such as benzoxazinoids and organic acids that influence microbial recruitment [50], these compounds generally exert weaker selective effects on the microbiome compared to the glucosinolates of *Brassicaceae* or the specialized rhizobia-legume symbioses. The absence of wheat-specific taxonomic signatures in our study is consistent with previous reports showing that cereal crops generally have a less pronounced effect on rhizosphere microbial community composition compared to crops producing specialized secondary metabolites [23]. This weaker plant-driven selection allows soil type and environmental factors to play more dominant roles in shaping the wheat rhizosphere microbiome. Under the extreme temperature stress experienced in our experiment, the already modest selective influence of wheat on its microbiome was likely further diminished, as stress conditions can alter root exudation patterns and reduce plants’ capacity to actively recruit beneficial microorganisms.

### 3.5. Practical Significance and Recommendations

The results of this study represent an important contribution to understanding the “lab-to-field transition” problem in PGPR application—the widely recognized gap between successful laboratory experiments and insufficient efficacy under field conditions. The causes of this phenomenon are diverse and include: environmental condition mismatches, competition with indigenous microflora, climatic factor influences, and strain-crop-soil interaction specificity.

Our research adds another important factor to this list: extreme weather conditions can completely nullify the potential beneficial effects of PGPR, especially when these effects are predominantly associated with improved nutrition rather than induction of systemic resistance to abiotic stresses.

#### Recommendations for Practical Application of Microbial Consortium

Based on the obtained results, the following recommendations can be formulated for practical application of microbial consortium:

1. Development of protective formulations (encapsulation, immobilization on carriers) to enhance strain survival under field conditions and ensure their prolonged activity.
2. Optimization of application protocols: combining pre-sowing seed treatment with repeated applications during critical plant development phases to maintain effective populations of introduced strains.
3. Incorporation into consortium of stress-protective strains capable of inducing systemic plant resistance to abiotic stresses (drought, extreme temperatures), rather than solely improving mineral nutrition. This is particularly relevant in the context of climate change and increasing frequency of extreme weather events.
4. Conducting multi-year experiments to assess cumulative effects on soil fertility, since many changes (e.g., in microbial community structure) may manifest in subsequent seasons.
5. Development of crop-specific consortium accounting for the peculiarities of rhizosphere interactions of particular plants. The observed differences among buckwheat, rapeseed, and wheat highlight the importance of such an approach.

## 4. Materials and Methods

### 4.1. Small-Plot Field Experiment Design

The field experiment was conducted from June to September 2024 at an experimental site with minimally disturbed soils in the Novosibirsk Region of Western Siberia, Russia (N: 54.8477639, E: 83.1584295). Site preparation included double plowing and leveling of the field surface to ensure uniform conditions. Metal frames (32 cm × 32 cm) were then installed to delineate individual experimental plots and prevent lateral water flow and potential migration of applied treatments during rainfall events. Guard rows of the corresponding crop species were planted around crop plots to minimize edge effects and interplot competition.

The experiment was conducted on two contrasting soil types representative of Western Siberian agriculture: chernozem and gray forest soil. Soil samples were collected from both soil types for agrochemical characterization and particle size distribution analysis prior to experiment establishment.

#### 4.1.1. Experimental Treatments

The experiment employed a randomized complete block design with four replicates to evaluate the effects of a synthetic microbial consortium (SMC) on three agricultural crops and soil properties under field conditions. Three crop species commonly cultivated in Western Siberia were selected: wheat (*Triticum aestivum* L.), buckwheat (*Fagopyrum esculentum Moench*), and rapeseed (*Brassica napus* L.).

Four treatment levels were established:

Untreated control (CK)—no fertilizers or SMC

Mineral fertilizers (MF)—half the recommended dose

Bacterial consortium (SMC)—microbial consortium application only

Combined treatment (MF + SMC)—mineral fertilizers plus microbial consortium

These four treatments were applied to plots with each of the three crop species on both soil types. Additionally, no-plant control plots were established with the same four treatments on both soil types to specifically assess SMC effects on soil properties independent of plant influence. This resulted in a total of 128 experimental units: 4 treatments × (3 crops + 1 no-plant control) × 2 soil types × 4 replicates.

#### 4.1.2. Seeding and Treatment Application

Seeding rates for each crop were based on recommendations for Western Siberian soil and climatic conditions, adjusted proportionally to the plot area of 0.1 m^2^. Seeds were sown manually to ensure uniform distribution across each plot at the following seeding rates: wheat—51 seeds, buckwheat—30 seeds, and rapeseed—24 seeds per plot. Based on preliminary greenhouse experiments showing no significant differences between application methods (seed inoculation, soil drench, repeated applications), a single soil drench application was selected for field implementation. According to the experimental design, treatments were prepared by dissolving the required amounts in 400 mL of water: mineral fertilizers (ammonium nitrate and potassium monophosphate) at 0.9 g per plot (half the recommended dose for cereal crops), bacterial consortium at 30 × 10^8^ CFU per plot, or a combination of bacterial consortium and mineral fertilizers. These solutions were applied in equal volumes to the root zone of emerged seedlings.

#### 4.1.3. Bacterial Consortium Composition

The experiment utilized a synthetic microbial consortium (SMC) consisting of five bacterial strains with complementary plant growth-promoting functions. The strains were isolated from the rhizosphere of various agricultural plants grown in different soil types across Russia as part of the citizen science project “Atlas of Soil Microorganisms of Russia” [51]. All strains are registered in the project database and deposited in the collection of the Laboratory of Molecular Genetics, Institute of Chemical Biology and Fundamental Medicine, Siberian Branch of the Russian Academy of Sciences (ICBFM SB RAS). Species identification was performed based on 16S rRNA gene sequences obtained through whole-genome sequencing analysis.

The consortium consisted of five bacterial strains responsible for key soil biological functions that promote adequate plant nutrition and growth: *Rahnella aquatilis* GMG_294, *Rothia* sp. GMG_009, *Stenotrophomonas lactitubi* GMG_165.2, *Burkholderia lata* GMG_664, and *Lelliottia nimipressuralis* GMG_355, exhibiting high phosphate-solubilizing and nitrogen-fixing activity, as well as high activity in the production of ammonium, auxin, and siderophores (Table 2). This functional diversity was intentionally designed to provide multiple mechanisms of plant growth promotion and potentially enhance consortium stability under field conditions. Cross-streaking assays on LB agar confirmed the absence of antagonistic interactions among the five strains, supporting their suitability for combination in a mixed consortium.

The five bacterial strains comprising the consortium were cultivated separately to maintain strain identity and enable precise quantification. Individual strains were grown in Luria–Bertani (LB) broth medium in 250 mL Erlenmeyer flasks containing 100 mL of medium. Cultures were incubated at 30 °C under aerobic conditions with constant agitation at 200 rpm on an orbital shaker for 48 h until reaching stationary phase. Bacterial cell density was determined by measuring optical density at 600 nm (OD_600_). For field application, the five cultures were mixed in equal proportions (1:1:1:1:1 *v*/*v*) and diluted with sterile physiological saline to a final total concentration of 10^8^ CFU/mL (approximately 2 × 10^7^ CFU/mL per strain). The consortium suspension was prepared fresh on the application day and used within 12 h.

#### 4.1.4. Climatic Conditions

**Figure 12:**
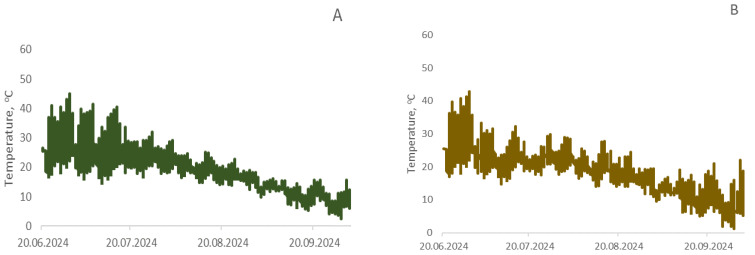

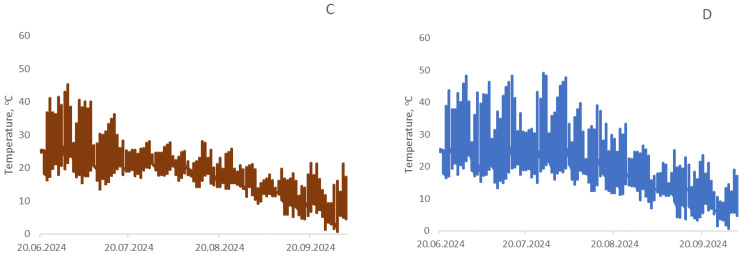
Temperature distribution in the root zone soil layer at a depth of 1 cm for wheat (**A**), buckwheat (**B**), rapeseed (**C**), and unseeded control (**D**) during the growing season.

Temperature was automatically recorded every 2 h throughout the entire growing season of the cultivated crops (Figure 12). Air temperature reached 43.8 °C during June–July 2024. On average, day and night temperature fluctuations ranged from 17.8 to 44.0 °C in June, from 11.7 to 41.2 °C in July, from 8.7 to 41.2 °C in August, and from −1.2 to 28.0 °C in September. Soil surface temperature in control plots without seed sowing was higher, which was particularly noticeable during the period when plants gained height and shaded the soil surface from solar radiation.

### 4.2. Agrochemical Analysis

Agrochemical characterization of soils and plant tissues was conducted according to standard protocols [52]. Soil extractable nutrients were measured as follows: nitrate nitrogen (N-NO_3_, mg/kg) extracted with 0.03 M K_2_SO_4_ and determined potentiometrically (Karpinsky-Zamyatin method); available phosphorus (P_2_O_5_, mg/kg) extracted with 0.03 M K_2_SO_4_ (Karpinsky-Zamyatin method); exchangeable potassium (K_2_O, mg/kg) extracted with 1 M CH_3_COONH_4_ (Maslova method). Exchangeable cations (Ca^2+^, Mg^2+^, and Na^+^) were determined by atomic absorption spectroscopy using a PerkinElmer spectrophotometer (PerkinElmer, Inc., Shelton, CT, USA). Plant tissue NPK content was determined following wet digestion, with subsequent analysis of exchangeable cations (K^+^, Ca^2+^, Mg^2+^, Na^+^, and Fe) by atomic absorption spectroscopy using a PerkinElmer spectrophotometer (PerkinElmer, Inc., Shelton, CT, USA). Soil samples for agrochemical analysis were collected at the end of the growing season (September).

### 4.3. Determination of the Yield of Buckwheat, Wheat and Corn

To assess the yield of crops grown as part of the experiment, the following parameters were recorded: plant height, mass of dry straw, mass of grain from the plot, mass of dry roots. The height of the plants was measured after 2, 4 and 6 weeks. The mass of dry roots, the mass of straw and the mass of grain were measured after the harvest in three months. For grain, the average weight from three plots was estimated (mean and SD), as well as the average amount of grain per plot, taking into account fallen grain, calculated as the sum of the weight of grain from three plots and fallen grain divided by three.

### 4.4. Soil DNA Extraction and NGS-Sequencing

#### 4.4.1. DNA Extraction

Total DNA from 0.5g soil was extracted using MagBeads FastDNA™ Kit for Soil (MP Biomedicals, LLC, Irvine, CA, USA) as recommended by the manufacturer. DNA quantity was estimated by Qubit 4.0 (Invitrogen/Life Technologies, Carlsbad, CA, USA).

#### 4.4.2. Sequencing of 16S rRNA Gene Libraries

The purified DNA isolates were amplified with primers previously developed by us Artik-FF (5′-GTGACTGGAGTTCAGACGTGTGCTCTTCCGATC**TCTACGGGAGGCAGCAG**-3′) and (Artik-FR 5′-ACTCTTTCCCTACACGACGCTCTTCCGATC**TGGACTACCGGGGTATCT**-3′) targeting variable regions V3–V4 of bacterial and archaeal 16S rRNA genes. PCR was carried out in a 25 μL reaction mixture containing 1 unit of Q5 Hot Start High-Fidelity DNA Polymerase and Q5 Reaction Buffer (New England Biolabs, Ipswich, MA, USA), 10 pM of each primer, 2 ng of DNA matrix and 2 nM of each dNTP and fluorescent dye SYBR Green. Amplification was performed in a CFX96 (Bio-Rad Laboratories, Inc., Hercules, CA, USA) under the following conditions: initial denaturation for 3 min at 96 °C then 40 cycles consisting of denaturation at 95 °C for 10 s, then annealing of primers at 55 °C for 30 s and subsequent elongation at 72 °C for 30 s The final elongation was carried out at 72 °C for 5 min. Visualization of PCR products was carried out by gel electrophoresis in agarose gels in the presence of ethidium bromide. PCR products were purified according to the recommended Illumina technique using AM Pure XP (Beckman Coulter, Inc., Brea, CA, USA). DNA concentration in solutions was determined using a desktop fluorimeter Qubit 4.0 (Invitrogen/Life Technologies, Carlsbad, CA, USA). To do this, we used the Qubit dsDNA HS Assay Kit according to the protocol. Enrichment was carried out using PCR (Invitrogen/Life Technologies, Carlsbad, CA, USA). A set of oligonucleotides Multiplex Oligos for Illumina Dual Index Primer Set 1 (New England Biolabs, Ipswich, MA, USA) was used as primers. PCR was carried out in a 25 μL reaction mixture containing 1 units Q5 Hot Start High-Fidelity DNA Polymerase and Q5 Reaction Buffer (New England Biolabs, Ipswich, MA, USA), 10 pM of each primer, DNA matrix and 2 nM of each dNTP and fluorescent dye SYBR Green. Amplification was performed in an CFX96 (Bio-Rad Laboratories, Inc., Hercules, CA, USA) under the following conditions: initial denaturation for 30 s at 98 °C then 12 cycles consisting of denaturation at 98 °C for 10 s, then annealing of primers and elongation at 65 °C for 75 s. The final elongation was carried out at 65 °C for 5 min. Then PCR products were purified according to the recommended Illumina technique using AM Pure XP (Beckman Coulter, Inc., Brea, CA, USA). To create a single pool of all libraries, we calculated how much of each library should be taken to get the same amount of DNA in nanograms. Calculations were made based on the concentrations of DNA in the enriched libraries measured using the Qubit 4.0 desktop fluorimeter (Invitrogen/Life Technologies, Carlsbad, CA, USA). To quantify amplicon libraries, the Real-Time PCR method was used with the addition of a TaqMan probe with a ROX dye label (Syntol, Moscow, Russia) and oligonucleotides complementary to the end sequences of libraries. As a quantitative standard, PhiX-control was used, diluted 10, 100, 1000 and 10,000 times. The concentration of the library pool was determined by the location of the fluorescence signal accumulation curve relative to the control samples. The 16S rRNA gene amplicons was sequenced in paired-end mode (2 × 301) with the Illumina MiSeq v3 (600 cycles) kit (Illumina, Inc., San Diego, CA, USA) on a MiSeq at the Sirius University of Science and Technology (Sirius, Russia).

### 4.5. Statistical Analysis

Comparing the groups for statistical differences in these data, we tested the significance using ordinary two-way ANOVA analysis and Tukey’s multiple comparisons test, as implemented in GraphPad Prism 10 (https://www.graphpad.com/features, accessed on 10 September 2025).

Analysis of 16S ribosomal RNA gene sequences was performed using QIIME 2 v.2023.7 following the protocol described in our previous study [53]. The description and visualization of alpha and beta diversity were performed using the phyloseq package in R [54].

## 5. Conclusions

The present study revealed a critical problem in the application of microbial consortium under field conditions: single-application strains failed to colonize the rhizosphere, while extreme temperature conditions (up to 43.8 °C) completely nullified the potential effects of both microbial consortium and mineral fertilizers. The absence of introduced strains in the soil throughout the growing season explains the lack of impact on plant morphometric parameters, despite transient changes in the structure of the indigenous microbiome. These results emphasize the necessity of developing protective formulations ensuring strain survival, optimizing application protocols with repeated treatments, and incorporaem ting thinto consortium stress-protective strains capable of inducing systemic plant resistance to abiotic stresses—particularly relevant in the context of climate change and increasing frequency of extreme weather events.

The observed crop-specific effects (significant increase in available phosphorus under rapeseed on chernozem, different patterns of microbiome changes under buckwheat, rapeseed, and wheat) indicate the potential of the microbiological approach, which can be realized under favorable conditions and with optimized application methods. Multi-year studies of cumulative effects from repeated consortium applications remain an important task for assessing long-term impacts on soil fertility and agricultural crop productivity.

## Figures and Tables

**Table 1:** The physico-chemical properties of the soils.

	pH	SEC, μs/cm	C_org., %	Ntotal, %	N-NO_3_, mg/kg	N-NH_4_, mg/kg	P_2_O_5_, mg/kg	K_2_O, mg/kg
Dark grey forest soil	7.1 ± 0.2	83.1 ± 0.8	7.2 ± 0.2	0.5 ± 0.1	3.5 ± 0.3	0.7 ± 0.3	1.2 ± 0.9	98.0 ± 0.0
Chernozem	7.8 ± 0.1	206.1 ± 0.9	10.3 ± 0.4	1.5 ± 0.01	4.5 ± 0.7	1.3 ± 0.3	11.3 ± 1.2	139.5 ± 2.1

**Table 2:** Plant growth-promoting traits of bacterial strains comprising the consortium.

No	Strain	Nitrogen Fixation, %	Ammonia Production, µmol/mL	Phosphate Solubilization, µg/mL	IAA Production, µg/mL	Siderophores Production, %
GMG_009	*Rothia* sp.	41.82 ± 17.77	8.44 ± 0	226.22 ± 78.26	1.49 ± 1.24	6.64 ± 27.29
GMG_165.2	*Stenotrophomonas lactitubi*	21.14 + 12.67	8.4 + 0.04	429.32 + 65.86	1.05 + 0.05	18.96 + 4.6
GMG_294	*Rahnella aceris*	3.68 + 0.16	2.38 + 0.25	544.5 + 106.61	51.11 + 0.8	39.45 + 11.87
GMG_355	*Lelliottia nimipressuralis*	6.3 + 0.4	5.12 + 0.53	292.04 + 42.27	127.28 + 7.39	27.29 + 3.88
GMG_664	*Burkholderia lata*	16.54 + 5.3	1.03 + 0.21	409.74 + 98.13	2.26 + 0.09	72.29 + 4.68

## Data Availability

The original contributions presented in this study are included in the article/Supplementary Material. Further inquiries can be directed to the corresponding author.

## Supplementary Materials

The following supporting information can be downloaded at: https://www.mdpi.com/article/10.3390/ijms262411814/s1.
