# Supplementary material for: Microbial Consortium Application Under Temperature Stress: Effects on the Rhizosphere Microbiome and Plant Growth
Source: Int J Mol Sci. 2025 Dec 7;26(24):11814. doi: 10.3390/ijms262411814 (PMC12732923; doi:10.3390/ijms262411814)
Supplement: Supplementary file 1 [file ijms-26-11814-s001.zip › ijms-4027863-supplementary.pdf]

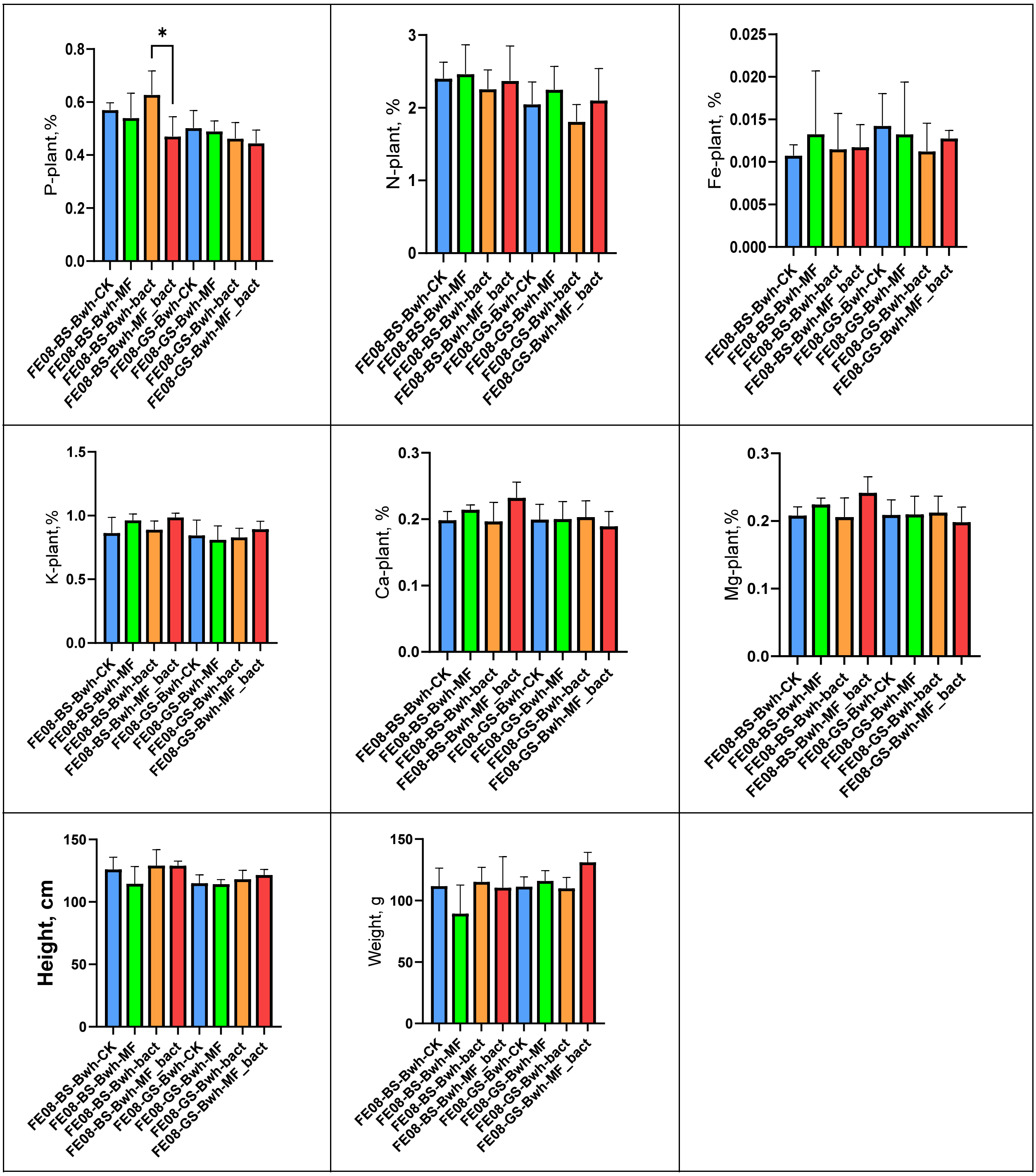

Figure S1. Plant morphometric characteristics and nutrient content of buckwheat: BS- chernozem, GS – grey soil, CK – without treatment, MF - mineral fertilizers, bact – microbial consortium, MF_bact – microbial consortium combined with mineral fertilizers

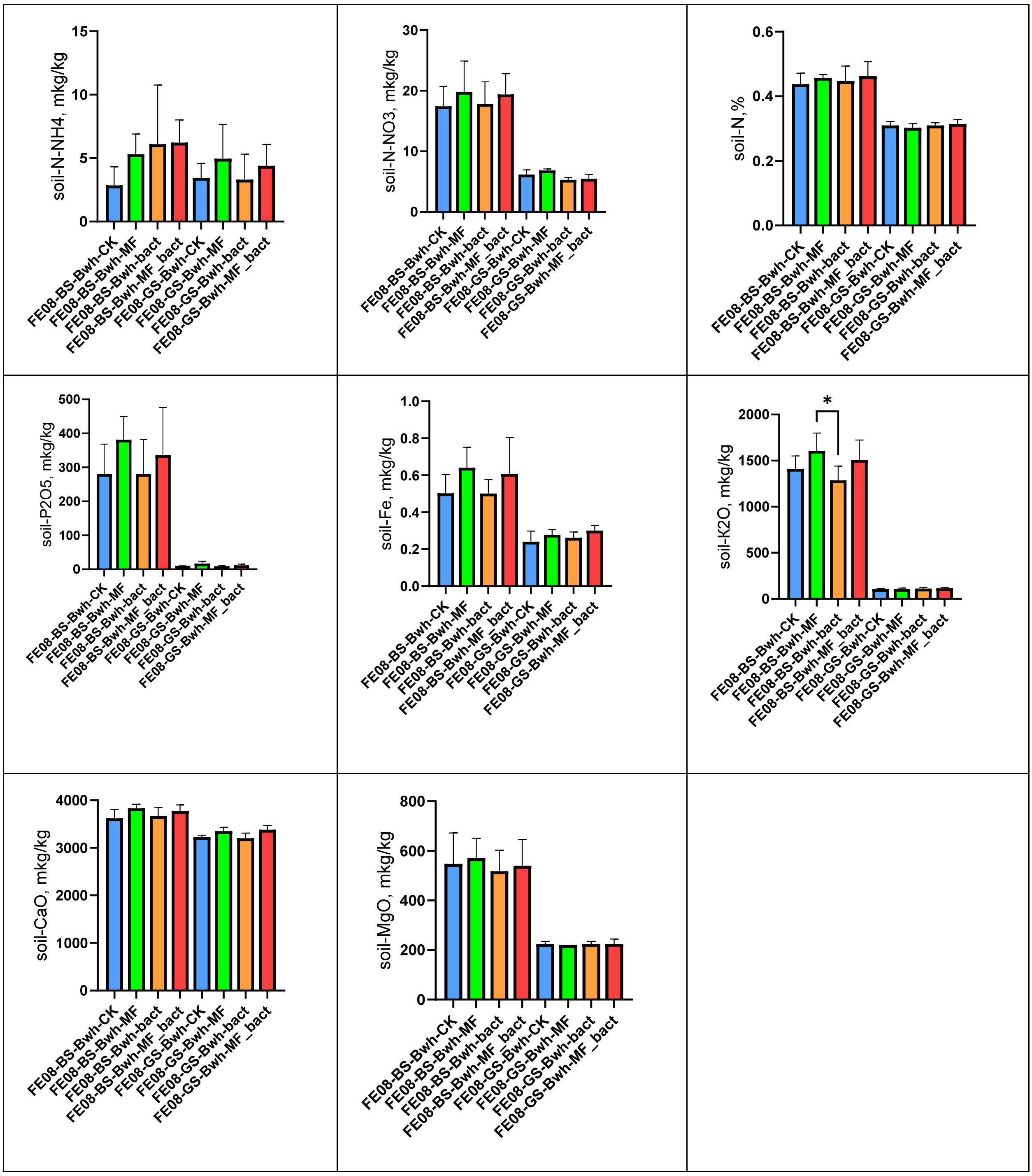

Figure S2. Elemental content in buckwheat rhizosphere soil: BS- chernozem, GS – grey soil, CK – without treatment, MF - mineral fertilizers, bact – microbial consortium, MF_bact – microbial consortium combined with mineral fertilizers

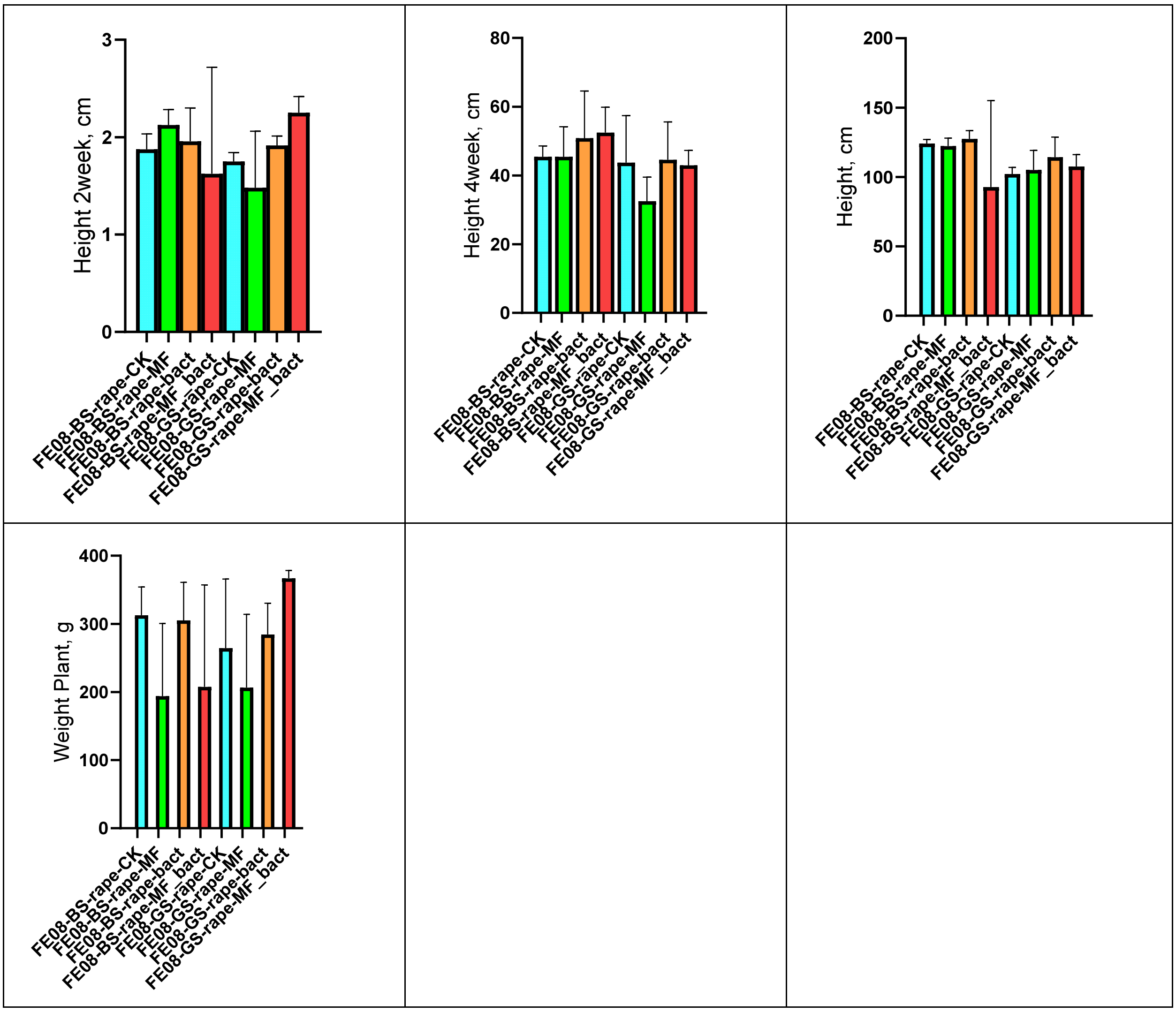

Figure S3. Plant morphometric characteristics of rapeseed: BS- chernozem, GS – grey soil, CK – without treatment, MF - mineral fertilizers, bact – microbial consortium, MF_bact – microbial consortium combined with mineral fertilizers

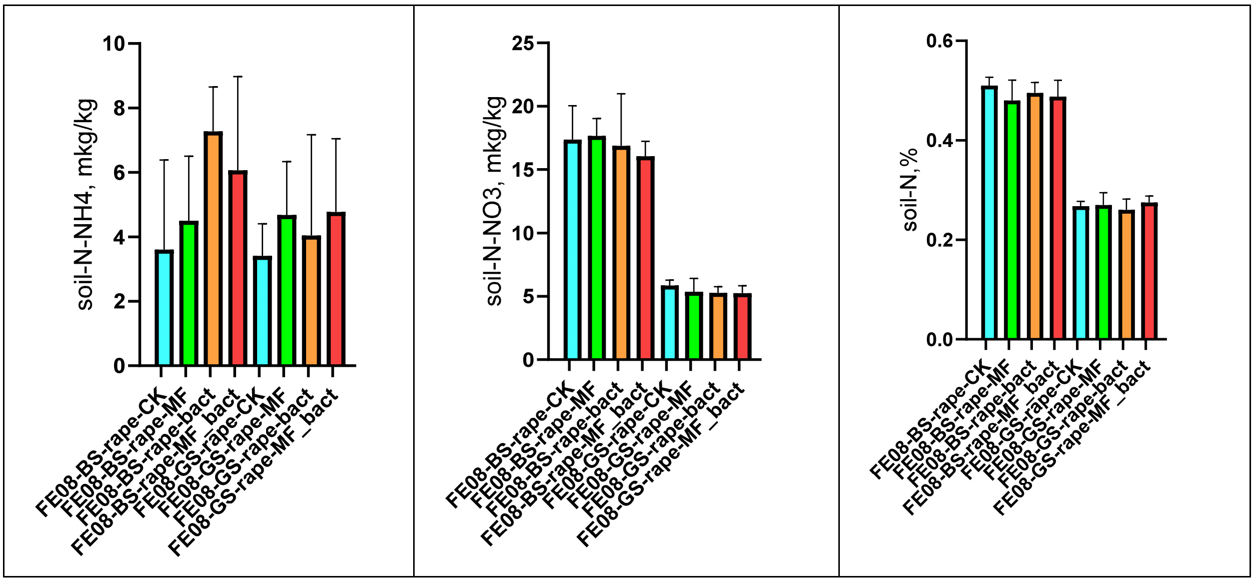

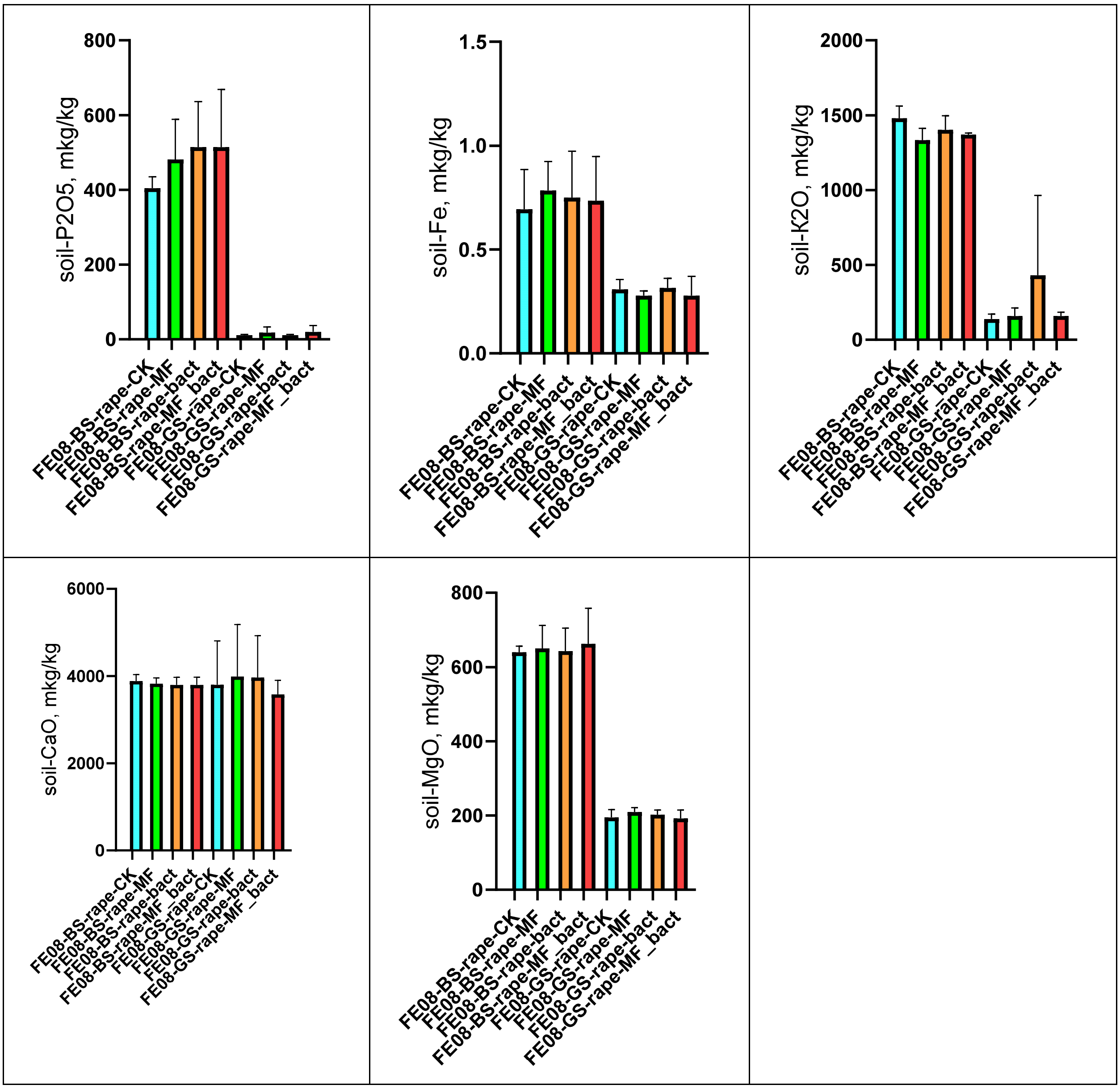

Figure S4. Elemental content in rapeseed rhizosphere soil: BS- chernozem, GS – grey soil, CK – without treatment, MF - mineral fertilizers, bact – microbial consortium, MF_bact – microbial consortium combined with mineral fertilizers

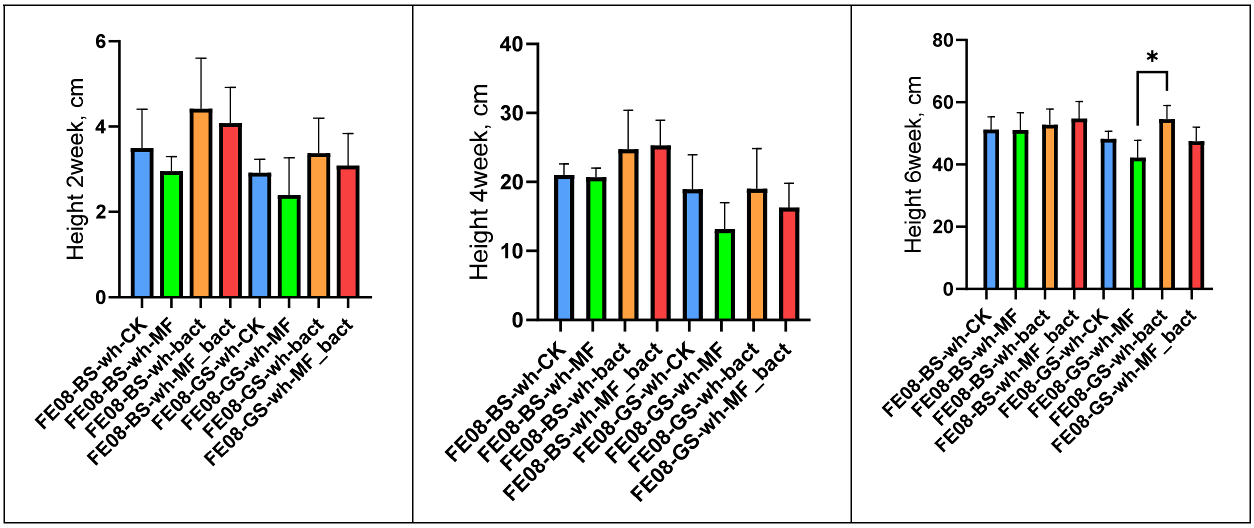

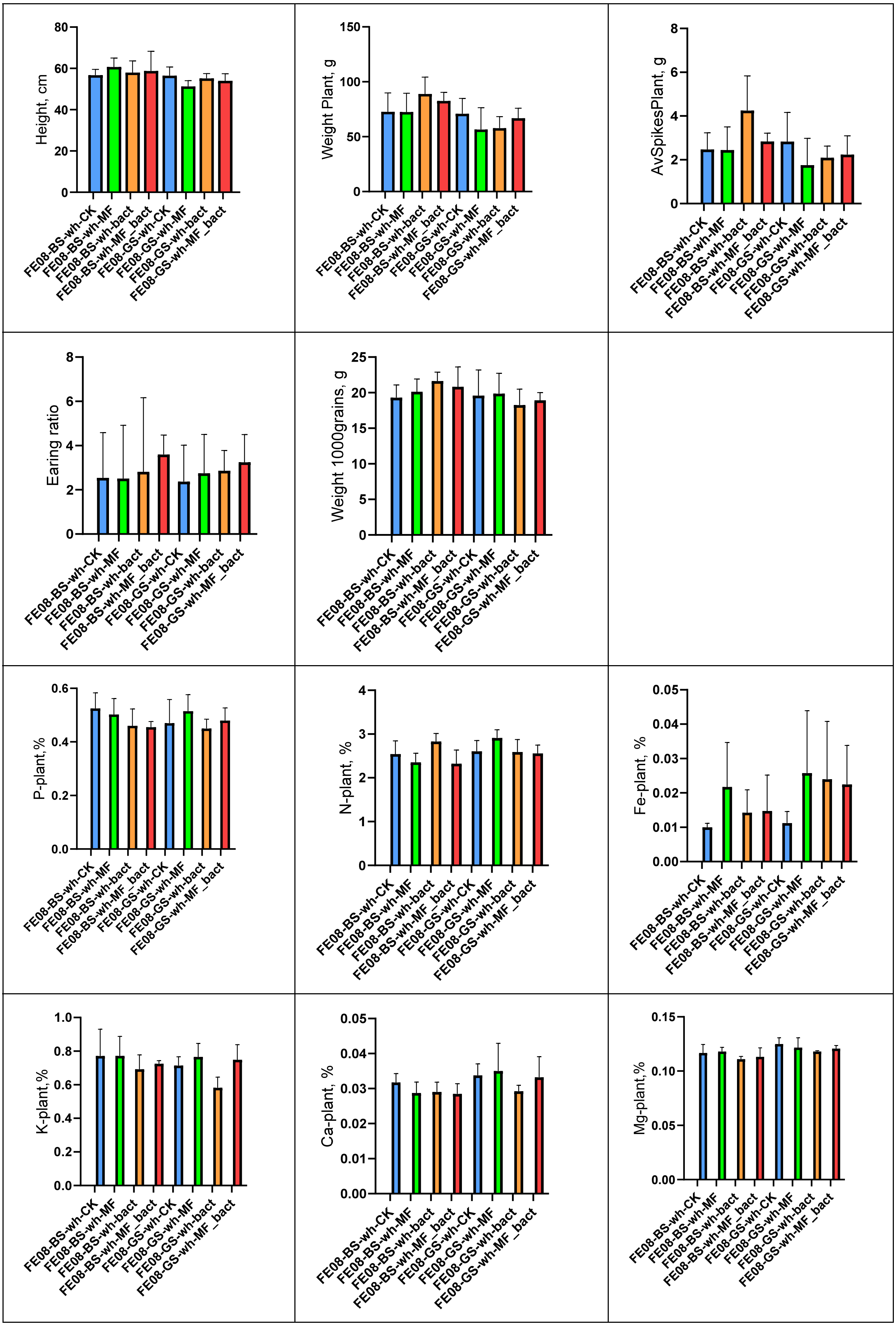
Height, cm
Weight Plant, g
AvSpikesPlant, g
Earing ratio
Weight 1000grains, g
P-plant,%
N-plant, %
Fe-plant, %
K-plant,%
Ca-plant, %
Mg-plant,%
FE08-BS-wh-CK
FE08-BS-wh-MF
FE08-BS-wh-bact
FE08-BS-wh-MF_bact
FE08-GS-wh-CK
FE08-GS-wh-MF
FE08-GS-wh-bact
FE08-GS-wh-MF_bact

Figure S5. Plant morphometric characteristics and nutrient content of wheat: BS- chernozem, GS – grey soil, CK – without treatment, MF - mineral fertilizers, bact – microbial consortium, MF_bact – microbial consortium combined with mineral fertilizers

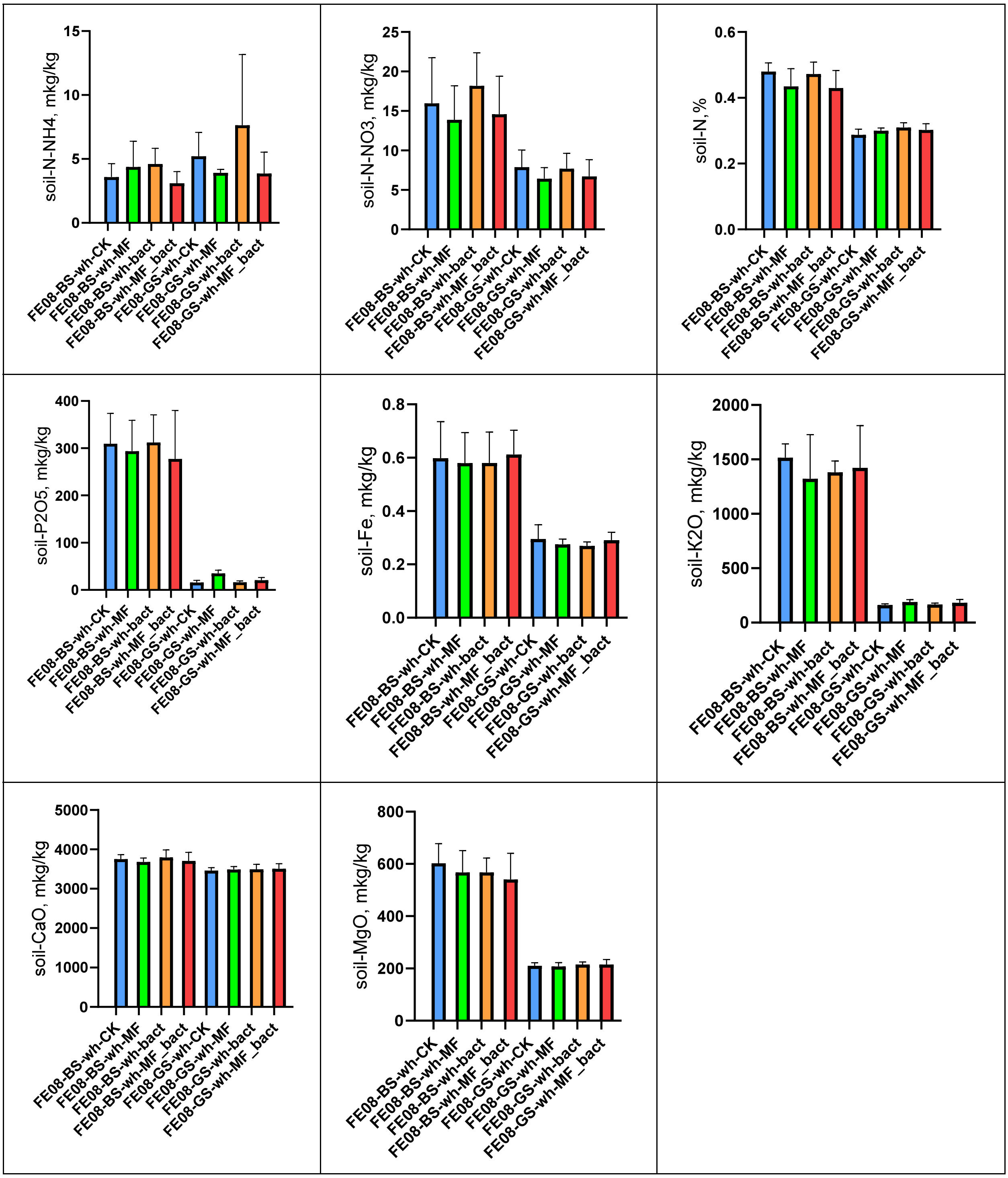

Figure S6. Elemental content in wheat rhizosphere soil: BS- chernozem, GS – grey soil, CK – without treatment, MF - mineral fertilizers, bact – microbial consortium, MF_bact – microbial consortium combined with mineral fertilizers
